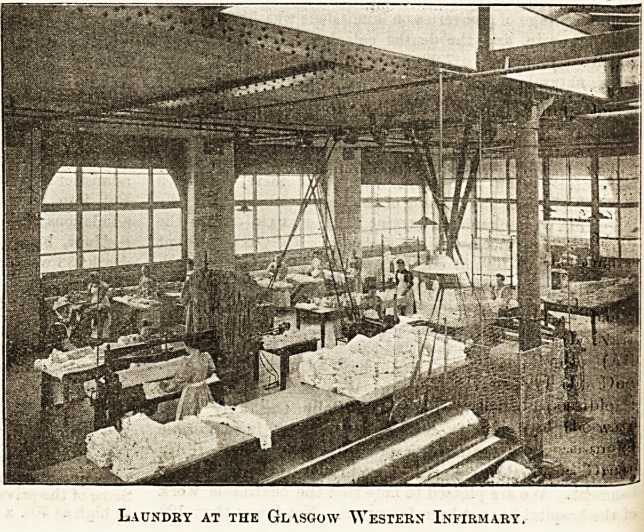# Practical Departments

**Published:** 1897-07-03

**Authors:** 


### PRACTICAL DEPARTMENTS.

### RECK'S CURRENT STEAM DISINFECTING APPARATUS.

Mr. Reck has made certain improvements in his apparatus

Fig. 1.—Stationary Disinfector with steam chart.

Fig. 2.—Section of disinfector with water jacket.

for the disinfection of bedding, clothing, &c. As is well known, this apparatus is one for the use of saturated current steam at a temperature of 220 deg. Fahr., or, if desired, of 230 deg. Fahr., for disinfecting purposes. The principle of the machine is that the steam enters the disinfecting chamber at the top, and drives out the air and condensed vapour at the bottom. The improvements which have been introduced consist of (1) an arrangement to prevent "priming" or the carrying over of particles of water along with the steam into the disinfecting chamber; and (2) an arrangement for retaining the heat of the chamber while the clothes within it are drying after the process of disinfection is complete; for, be it observed, the disinfection of clothes by steam of necessity involves the condensation of a certain amount of water in their interstices whatever apparatus is used. The first object is attained by placing a cylindrical steam chest (Fig. 1) at a considerable height above the boiler—above the disinfecting chamber, in fact—so that the steam is drawn off above the area of splashing. There is an additional advantage in this arrangement in that the cylinder acts as a hot water reservoir for the boiler, which thus does not require feeding so often as would otherwise be the case. The second object is met by surrounding the disinfecting chamber with a "water jacket" (Fig. 2), by which a considerable amount of heat is retained even after the steam is turned off—enough, in fact, to dry the clothes when air is admitted by slightly opening the door.

### LAUNDRY CONSTRUCTION.

We have received a series of eight beautifully executed photographs of the new laundry at the Glasgow Western Infirmary. The designers appear to have had exceptional facilities with regard to space and situation for making the building a model one of its kind, and the ventilation difficulty, one of the most serious to be encountered in premises of this description, can have no existence in these light and airy rooms, where the windows, as in the illustration of the ironing-room we reproduce, occupy nearly half the entire wall space, and are yet further supplemented by spacious skylights. The white tiling of the walls gives an additional air of brightness and cleanliness to the structure. The premises for the washing of hospital and staff linen are of course entirely distinct, and are each completely fitted with the best modern machinery. Both staff and hospital washhouses are admirably planned, and contrast very favourably indeed with the crowded and somewhat oppressive aspect of many old-fashioned institution laundries. It is obvious that speed, efficiency, and, therefore, economy are promoted by ensuring that the work shall be done under the best possible sanitary conditions, and it will be interesting to learn the effect on the laundry expenditure of the infirmary of this new and complete undertaking.

Laundry at the Glasgow Western Infirmary.

## Figures and Tables

**Fig. 1. f1:**
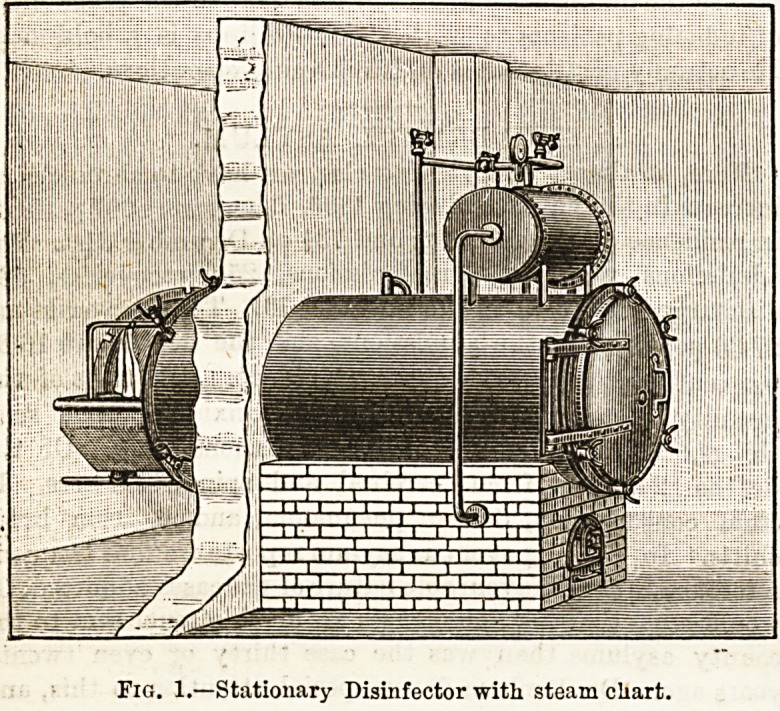


**Fig. 2. f2:**
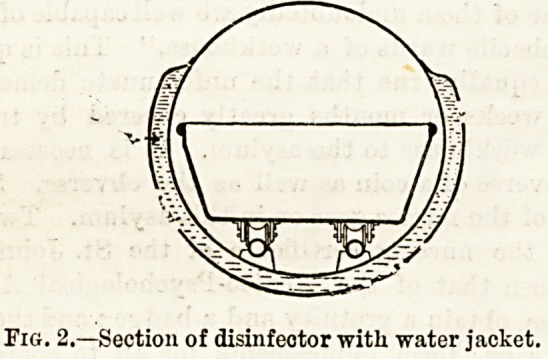


**Figure f3:**